# Conservation of fish diversity in protected sites and adjacent fishing areas of the Southern Mexican Pacific Ocean

**DOI:** 10.1371/journal.pone.0324155

**Published:** 2025-06-04

**Authors:** Georgina Ramírez-Ortiz, Omar Valencia-Méndez, Luis Hernández, Tania González-Mendoza, Andrés López-Pérez

**Affiliations:** 1 Laboratorio de Ecología Funcional & Conservación Marina, Instituto de Ciencias del Mar y Limnología, Universidad Nacional Autónoma de México, Mazatlán, Sinaloa, México; 2 Laboratorio de Esclerocronología, Ecología y Pesquerías de la Zona Costera, Departamento de Ecología Marina, Centro de Investigación Científica y de Educación Superior de Ensenada, Ensenada, Baja California, México; 3 Laboratorio de Sistemas Arrecifales, Departamento Académico de Ciencias Marinas y Costeras, Universidad Autónoma de Baja California Sur, La Paz, Baja California Sur, México; 4 Laboratorio de Arrecifes y Biodiversidad/Ecosistemas Costeros, Departamento de Hidrobiología, Universidad Autónoma Metropolitana-Iztapalapa, Iztapalapa, Ciudad de México, México; MARE – Marine and Environmental Sciences Centre, PORTUGAL

## Abstract

The global decline in marine biodiversity is accelerating, prompting Mexico’s government to establish marine protected areas to regulate human activities. The objective of this study was to test whether there were differences in fish taxonomic and functional diversity among protected reef sites within Parque Nacional Huatulco and adjacent non-protected (NP) zones, as well as whether the temporal trends in fish diversity, density, and biomass of commercial and non-commercial species differed in relation to these human use levels. It was hypothesized that fish functional diversity would be similar among protected and NP sites, given that fishing and tourism activities persist at protected sites. Concurrently, taxonomic and functional diversity decline was predicted, associated with decreasing commercial species due to increasing human activities in both zones. We collected data from underwater monitoring conducted by SCUBA divers (2006–2020) at 20 coral and rocky reef sites to calculate taxonomic and functional diversity metrics based on six biological traits. Functional diversity metrics were employed to analyze the resemblance among human use levels. Additionally, ecological indicators were incorporated as response variables in linear models to assess temporal changes. The results demonstrated functional diversity resemblance among human use levels, as well as temporal stability in Simpson’s dominance index and significant increases in fish species richness and density at both zones. For protected sites, significant changes in fish functional diversity (increase in functional richness and decrease in divergence and originality), could indicate positive effects of protection, such as functional redundancy increases and the capacity to maintain reef functions over time. Commercial and non-commercial species exhibited stability or increase in density and biomass at both protected and NP sites.

## Introduction

The global decline in marine species richness and abundance is occurring at an accelerated pace, which could potentially compromise the resilience of marine ecosystems due to the reduction of functions [1,2]. To address this biodiversity loss, different conservation mechanisms have been implemented, including the establishment of Natural Protected Areas (NPAs) [3]. In Mexico, there are six categories of biodiversity protection (e.g., national parks, biosphere reserves, sanctuaries, etc.), encompassing 232 NPAs and covering a total area of 980,007.19 km^2^ [4,5]. Of this area, 23.78% consists of coastal and marine territories, which include 40 areas that can be considered Marine Protected Areas (MPAs). These MPAs have been established to preserve species and enhance ecosystem resilience through different strategies, from full to minimal protection from abatable threats (e.g., fishing) [6].

Grorud-Colvert et al. [6] identified four elements as defining protection types and activities, conditions for success, and outcomes: a) Stages of establishment of an MPA (proposed by the government, designated by law, implemented with regulations, and actively managed); b) Levels of protection from activities (fully protected with no impact from extractive activities, highly protected with minimal impact, lightly protected with moderate impact, and minimally protected with high impact); c) Conditions for effective and equitable MPA planning, design, governance, and management; and, d) Outcomes of the MPA that depend on the stage, level, and conditions to succeed.

Implementing fully protected areas (i.e., areas where no extractive or destructive activities are allowed and all abatable impacts are minimized) has demonstrated a notable increase in fish diversity compared to fishing zones [7–9]. Furthermore, these benefits can increase with key MPA characteristics, such as the duration of protection (> 10 years), the isolation of the MPA (MPAs with reef habitat surrounded by deep water or large expanses of sand tend to show the greatest benefits), the effective implementation of regulations, and the size of the protection polygon (> 100 km^2^) [10]. In addition, larger areas will sustain focal species within their boundaries, facilitating growth to maturity and reproductive potential. Ultimately, this will contribute to stock recruitment and regeneration of non-protected adjacent zones through larval dispersal and spillover of individuals at different life stages [10,11].

In contrast, reports for multi-use marine protected areas (MUMPAs; i.e., areas that aim to maintain biodiversity while allowing sustainable extractive activities) have indicated that there have been no significant increases in the diversity of fish assemblages, regardless of whether the taxa in question are commercial or non-commercial [11–13]. Since the principal aim of protection by some of the MUMPAs is to preserve (maintain) natural resources followed by the restoration of critical habitats [12,13], conservation outcomes such as stability or increase in biological diversity could be considered ecological benefits of this protection scheme. These findings have prompted intense debate about the advantages of establishing MUMPAs to conserve ecosystems in developing countries, where complex socio-ecological systems strive to gain the designation of large fully protected areas [12,14,15].

Understanding the ecological effects of protection by MPAs on both commercial and non-commercial species requires long-term investigations into the dynamics of fish communities in the Mexican Pacific, which have been constrained, particularly regarding the comparative advantages of MUMPAs and non-protected (NP) zones. Previous research has concentrated on protected areas in the Gulf of California, thereby creating a gap in knowledge regarding the southern Mexican Pacific region [13,16,17]. For example, Cabo Pulmo (23°N), Baja California Sur, which could be considered the only fully protected area in the country (its core zone comprises 99.6% of the protection polygon), demonstrated insignificant changes in fish taxonomic and functional diversity over 13 years (2005–2017) [18]. These findings were interpreted as indicative of ecosystem resilience, given that adjacent MUMPAs, Loreto (26°N) and Espíritu Santo (24°N), showed temporal declines in species richness, translated into decreases in functional richness. These temporal changes were associated with some disturbances, including increases in fishing, tourism, and sea surface temperature [18].

For the southern Mexican Pacific, a temporal trend analysis conducted by López-Pérez et al. [19] assessed the influence of environmental factors on fish diversity from 2006 to 2009 at 31 reef sites along the Oaxaca coast, including sites inside Parque Nacional Huatulco (MUMPA established in 1998) and NP sites. The results demonstrated notable discrepancies in fish diversity across sites, seasons, and years. These variations were linked to habitat characteristics (rocky ecosystems exhibited a higher abundance of fish than coral reefs) and sea temperature fluctuations (richness and abundance were greater during the upwelling season). Additionally, the study indicated that coral reef degradation and overfishing were prevalent in the region. This was evidenced by the dominance of non-commercial taxa (e.g., Pomacentridae and Labridae) over commercial fish families such as Lutjanidae, Serranidae, and Carangidae. Nevertheless, the impact of MUMPA regulations, particularly on fish community structure and dynamics was not considered.

Given its geographical location, Parque Nacional Huatulco is regarded as a keystone MUMPA, serving as a genetic reservoir for threatened, endemic, and ecologically relevant fish species. The rocky and coral reef ecosystems inside this MUMPA are considered the best-preserved in the region, this contributes to its critical ecological role [20,21]. A recent review of the literature and underwater monitoring data has identified 196 species of fish within this MUMPA [22]. Furthermore, fish diversity studies based only on underwater monitoring data, report species richness ranging from 60 to 89 fish taxa [23,24]. This is comparable to the values observed in temporal studies conducted in the central Mexican Pacific (80 species) [25] and the Gulf of California (99 species) [26].

MPAs are widely recognized for their role in conserving biodiversity and promoting the recovery of fish communities. However, the effectiveness of different levels of protection, particularly in MUMPAs, remains underexplored. While fully protected areas strictly prohibit extractive and destructive activities, MUMPAs allow different resource use, balancing conservation goals with sustainable human activities [27]. Understanding how these management strategies influence fish diversity is crucial, especially in regions facing high anthropogenic pressure. In the case of Parque Nacional Huatulco, there has been limited analysis of the long-term effects of protection on biodiversity. Although the MUMPA management program was formally implemented [28], the impact of its regulations on the local fish community has not yet been evaluated. This study seeks to fill that gap by compiling and analyzing a long-term dataset to assess temporal changes in fish communities as a response to protection by Parque Nacional Huatulco. We also included the analysis of adjacent fishing areas as a reference to the fish diversity and its temporal changes at the regional level. Specifically, we aimed to investigate whether there are differences in fish taxonomic and functional diversity between MUMPA and NP sites and to explore how temporal fish diversity, density, and biomass trends—particularly for commercial and non-commercial fish species— differed to these human use levels.

We hypothesized that, due to ongoing fishing and tourism activities at protected sites, fish functional diversity metrics would be similar between MUMPA and NP sites. Additionally, we predicted a decline in both taxonomic and functional diversity, driven by decreasing populations of commercial species in response to increasing human pressures across both protected and NP zones. Overall, this study aims to evaluate whether protection by a MUMPA offers potential benefits for reef fish assemblages in the southern Mexican Pacific, contributing to the broader understanding of MUMPA’s effectiveness in tropical regions.

## Materials and methods

### Study area

**Fig 1:**
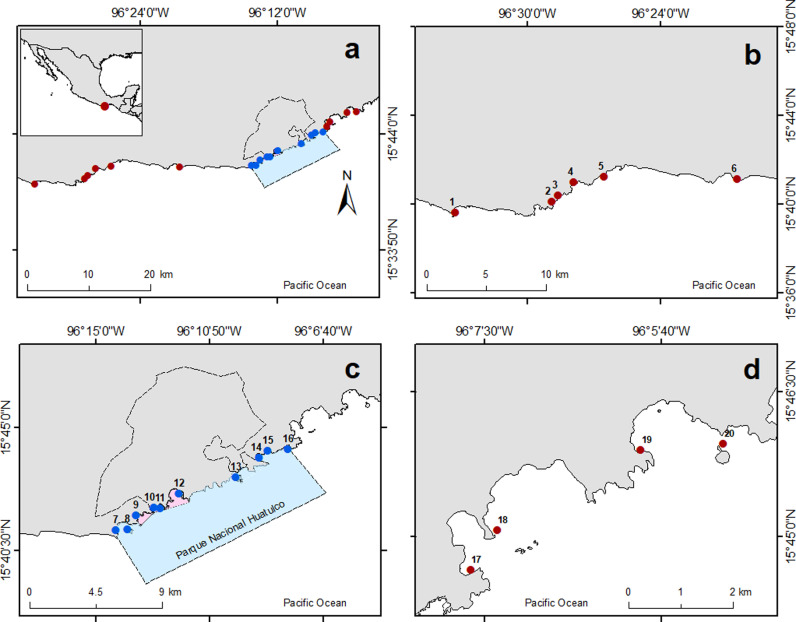
a) Study area in the southern Mexican Pacific. It includes two distinct groups of sites: b and d) ten non-protected sites (NP: 1-6 and 17-20), indicated by red dots, and c) ten protected sites within Parque Nacional Huatulco (MUMPA: 7-16), marked with blue dots. The protected area borderlines are outlined with a black line. The name of each site is included in Table A in S1 Text.

Bahías de Huatulco is situated on the coast of the Oaxaca state in southern México (Fig 1). It was designated as a Ramsar site (2003) and a Man and the Biosphere site (2006) by international agencies due to its ecological and cultural significance [28,29]. The Mexican government designated in 1998 a terrestrial and marine portion (area = 118.9 km^2^) of this region as a protected area. Subsequently, a management program was published, establishing the objective of Parque Nacional Huatulco, which is to preserve biodiversity and habitats, and promote their conservation through the sustainable use of its natural and cultural resources [28]. In accordance with this goal, the Parque Nacional Huatulco is subdivided into four discrete management zones, each designated according to its specific conservation objectives. The protected zones encompass areas with minimal human disturbance or fragile ecosystems, where only conservation efforts and scientific research are permitted. Restricted zones comprise well-preserved areas that are conducive for research and low-impact tourism. Traditional use zones are designated for areas with a history of resource use, which permit the extraction of certain species and aquaculture. In addition, sustainable use zones allow commercial and sport fishing and other activities permitted in the other zones [28]. The different types of natural resource use implemented in each zone, make Parque Nacional Huatulco qualify as an MUMPA.

The National Park encompasses five of the nine principal bays in the Huatulco region: San Agustín, Chachacual, Cacaluta, Maguey, and Órgano. These are distinguished by their rocky habitats and coral reefs, with an estimated average coral cover exceeding 60% across all sites reported in 2017 [28,30]. The marine habitats within the study area exhibit a sea surface temperature characteristic of tropical regions, with a mean value exceeding 28°C and minimal fluctuations of less than 2°C. The thermocline is relatively shallow and stable, occurring at depths between 20 and 40 m, with an average salinity of 34 ppt [31]. Given its proximity to the Gulf of Tehuantepec, the area experiences an upwelling event from October to April, causing a significant impact on the productivity and dynamics of the surrounding shallow habitats [32].

### Fieldwork and data analysis

To assess temporal changes in the fish community at MUMPA and NP sites, we collated data from underwater monitoring programs conducted between 2006 and 2020 (except for 2014 when sampling was not performed). Biological monitoring was conducted in various months, with surveys varying between years due to funding availability (Table A in S1 Text). The year was divided into two seasons: cold (January-June) and warm (July-December), based on historical data gathered from satellite images monthly from 2003 to 2019 using a Moderate Resolution Imaging Spectroradiometer (MODIS) Aqua sensor (Fig A in S1 Text). The season was incorporated as a random factor in temporal linear mixed-effect models (LMMs).

Fish surveys were conducted using SCUBA gear at depths between 2 and 10 meters, in 20 sites (MUMPA = 10 sites; NP = 10 sites), considered as rocky reefs except for site 7 (San Agustín inside the MUMPA) and the NP site 17 (La Entrega; Fig 1) that constitute coral reefs. At each site, two to ten belt transects were conducted by the same group of four divers primarily at the same time of day and tidal state; the number of sampling units at each visit depended on the environmental conditions. During the 2006–2013 and 2016–2020 periods, each transect was 20x4m (80m^2^), while during the 2015 period, data were gathered in transects of 25x4m (100m^2^). In total, 945 sampling units were conducted, from which species richness (number of species per sampling unit) and abundance (number of individuals per sampling unit) were calculated. From 2012 to 2020, individual size was considered in the fish censuses, thereby enabling the estimation of fish biomass per sampling unit using the length-weight relationship. The weight of each fish was calculated using the following formula: Weight = a*Length^b^, where *a* and *b* are the coefficients obtained from FishBase [33]. To facilitate comparison between sites, abundance and biomass per sampling unit were standardized (divided by 80 or 100, depending on the transect area) to obtain density (individuals/m^2^) and biomass (g/m^2^). The Simpson’s dominance index (D), which indicates the probability of two randomly chosen individuals being associated with the same species, was calculated using the species richness and density data [34].

The fish species registered in the sampling units were grouped in functional entities (FEs) according to six biological traits (Table B in S1 Text) that have been previously used in studies of this kind: size, mobility, period of activity, aggregation, position in the water column and diet [18,35,36]. Utilizing this information, we constructed a matrix of categorical variables, which were transformed into a numerical matrix to facilitate paired comparisons between species. This was achieved with the Gower dissimilarity coefficient, as implemented by the “daisy” function within the cluster R package [37].

From the dissimilarity matrix, an ordination of species in a reduced-dimensional space was performed using the “pcoa” function of the ape R package [38], which is referred to as Principal Coordinate Analysis (PCoA). To evaluate the extent of distortion between the initial trait distance matrix (species pairs) and the distance matrix after dimensionality reduction (PCoA axes), we employed the methodology proposed by Maire et al. [39]. Based on the aforementioned analysis, the first five axes (Fig B in S1 Text) were selected to calculate functional indices, in addition to two weight matrices. First, the average density per species at each human use level (n = 2; MUMPA and NP sites) was used to represent in two-dimensional graphs (PCoA1-PCoA2 and PCoA3-PCoA4), the overlap between MUMPA and NP fish assemblages. Second, the density per species at each sampling unit was considered (since 193 transects had less than six FEs, 502 sampling units for MUMPA and 250 sampling units for NP sites were used) to assess temporal diversity trends of fish assemblages at both zones. To ensure that differences in sample size did not influence species richness patterns, rarefaction curves were performed using the vegan R package (Fig C in S1 Text) [40].

The functional indices considered in this study were functional richness (FRic: the volume occupied by all FEs within the functional space), functional divergence (FDiv: the proportion of total density supported by FEs with the most extreme trait values within the assemblage), and functional originality (FOri: the isolation of a FE in the functional space occupied by a given assemblage) [35]. The development of their graphs was conducted using the “multidimFD’‘ function [41], in conjunction with the tidyverse [42], ggConvexHull [43], emestreeR [44], patchwork [45], and ggplot2 [46] R packages.

The functional indices values per sampling unit, in addition to species richness, density, and D, were employed as response variables in LMMs for both human use levels to analyze the effect of protection (fixed factor) despite the natural variability of the data (random factors), since surveys were performed in different years (2006–2020), seasons (warm or cold), and sites (locations> 200 m apart). The use of LMMs offers several advantages, as they allow us to infer the effect of random variables by replacing their individual parameters with a single intercept [47]. In this context, T and P indicate whether a random variable included in the model has a significant effect on fish ecological indicators, while the random standard deviation (RSD) measures the variability in the random effects [47]. Higher RSD values indicate greater variation in the effect of the random variable, suggesting a stronger influence of the analyzed factor on the model [47]. Additionally, separated LMMs for MUMPA and NP sites were performed, to assess temporal changes, thus year was considered as a fixed factor, as long as season and site were included in the models as random variables. This approach was undertaken to account for intra-annual variation and spatial heterogeneity [47]. The models were conducted with the “lmer” function of the lme4 R package [48], the results were obtained using the “summ” function of jtools [49], and the estimates were graphed using the ggplot2 R package [46]. To address the impact of specific climatological events, such as the El Niño–Southern Oscillation in 2009–2010 and 2015–2016 [50], we conducted a comparative analysis of the six ecological indicators between sampling years using ANOVAs implemented in the stats R package.

To assess temporal trends in mean density (2006–2020) and biomass (2012–2020) of commercial and non-commercial fish (both metrics were transformed using the log X + 1 transformation), we employed linear models for MUMPA and NP sites. Finally, to identify representative species in terms of density and biomass for both zones, we carried out one-way SIMPER analyses (Bray-Curtis dissimilarity coefficient) using PRIMER v6 [51].

The relevant authorities at the Parque Nacional Huatulco, which is under the jurisdiction of the Comisión Nacional de Áreas Naturales Protegidas approved the field surveys. Before 2014, the MPA authorities only required notification of the performance of field surveys. Permits for the removal or manipulation of specimens were not required, as our data set comprises visual censuses, however, throughout the study, two scientific collection permits were obtained from the Comisión Nacional de Pesca: PPF/DGOPA-035/15 and PPF/DGOPA-116/17.

## Results

A total of 84 conspicuous fish species were recorded on the Oaxaca coast, 78 species grouped in 65 FEs were present at MUMPA sites, while 73 species grouped in 63 FEs were registered at NP sites. According to the rarefaction curves (Fig C in S1 Text), the species richness was well represented in both areas, despite the differences in the sampling effort, since NP sampling units were around half compared to the number performed at MUMPA sites (Table A in S1 Text).

**Fig 2:**
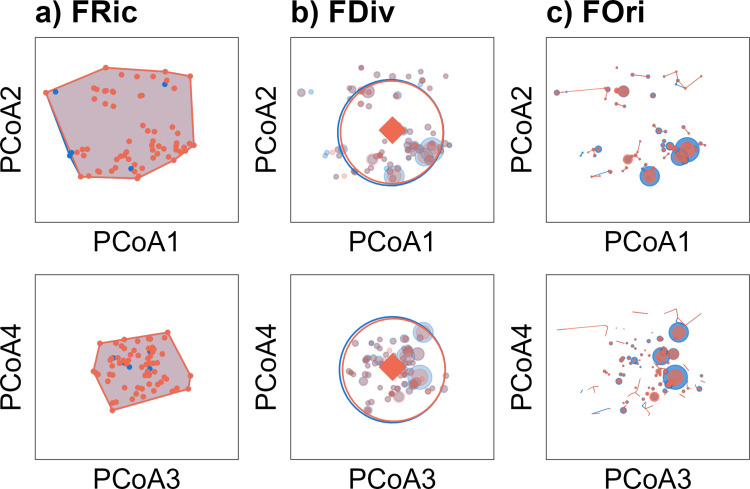
The functional structure of fish among protected (MUMPA: blue) and non-protected (NP: orange) sites of the Oaxaca coast are illustrated. The first four axes of the PCoA are used to plot functional indices, which are defined as follows: a) Functional richness (FRic): volume occupied by functional entities; b) Functional divergence (FDiv): proportion of total density supported by functional entities with extreme traits; c) Functional originality (FOri): isolation of functional entities within the functional space.

There was a high degree of overlap in fish functional indices between MUMPA and NP sites, particularly in the convex hull of FRic (Fig 2a) and the centroid and ellipse of FDiv (Fig 2b). These results indicate similarities in the presence and proportion of the total density supported by the FEs with the most extreme traits. Minor discrepancies were noted in the FDiv and FOri indices (Figs 2b and 2c), which were linked to elevated concentrations of specific FEs at the MUMPA locations (e.g., medium size, mobile within-reef, diurnal, solitary, bentho-pelagic, planktivores, and invertivores).

**Fig 3:**
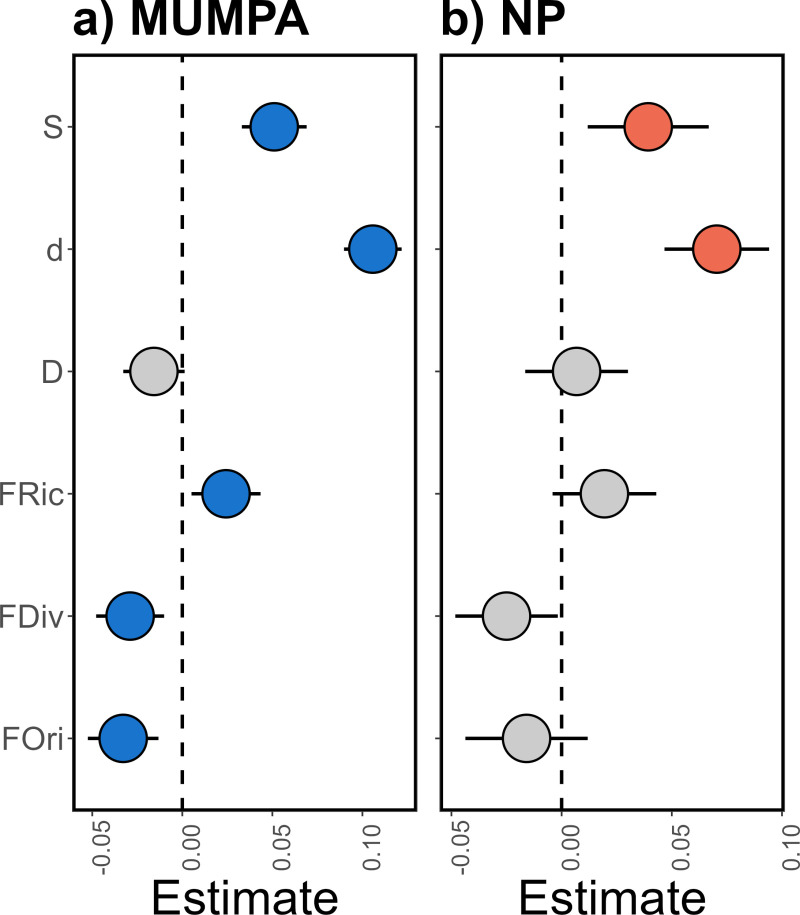
The standardized coefficients (mean ±  95% confidence interval) of Linear Mixed Models for fish ecological indicators calculated for protected (MUMPA: blue) and non-protected (NP: orange) sites of the Oaxaca coast are presented. The indicators are: species richness (S), density (d), Simpson’s dominance index (D), functional richness. (FRic), functional divergence (FDiv), and functional originality (FOri). The colored circles indicate statistically significant changes in the index, while the gray circles indicate non-significant changes over the study period (2006-2020).

The resemblance among human use levels was corroborated by LMMs, which exhibited a non-significant effect of protection for any of the indices considered in the study (Table C in S1 Text). This indicated that temporal trends (2006–2020) at MUMPA and NP sites were similar, as the increase in fish species richness and density were observed at both zones, with slight higher estimated values at sites within the MUMPA compared to NP sites (Table D in S1 Text). Nevertheless, density increases within species were similar at both zones, since Simpson’s dominance index (D) did not exhibit significant changes through the study period (Fig 3).

For MUMPA sites, FRic also showed a significant increase (Table C in S1 Text), indicating that the prevalence of FEs with extreme traits (large size, very mobile, nocturnal, and gregarious piscivores) is on the rise at protected sites, particularly in 2015 when it exhibited considerable distinctions due to the highest values (Fig D in S1 Text). However, these FEs (e.g., Carangidae, Lutjanidae, and Serranidae) could be considered rare, given that the FDiv and FOri significant decreases indicate that the majority of the observed increases in density occurred at FEs situated in proximity to the centroid of the functional space (Fig 3a). Therefore, the conservation of common (medium size, mobile within-reef, diurnal, solitary, bentho-pelagic, planktivores, and invertivores) reef species populations, represented by families such as Acanthuridae, Labridae and Pomacentridae, was primarily facilitated by the establishment of the MUMPA (Fig 3a).

The three analyzed functional indices showed maintenance throughout the study at NP sites, despite the significant increases in species richness and density. This is likely attributable to the appearance of redundant species and a general increase in density across the fish’s functional structure (Fig 3b). Concerning the data comparison from different years (Table D in S1 Text), NP sites exhibited markedly elevated values for species richness and density in 2015 (Fig E in S1 Text), corresponding with the El Niño–Southern Oscillation event of 2015–2016.

In addition to temporal changes (analysis between years), which showed the highest RSD values, our analysis demonstrated that both site and season also had significant effects on most of the indices (Table D in S1 Text). Specifically, the site variable showed higher RSD values, indicating that spatial variation had a stronger effect on fish diversity than seasonal changes. These factors were accounted for by the LMMs (Table C in S1 Text), but caution is needed when interpreting these results, as our data is unbalanced (i.e., there is unequal replication across years, sites, seasons, and human use levels).

**Fig 4:**
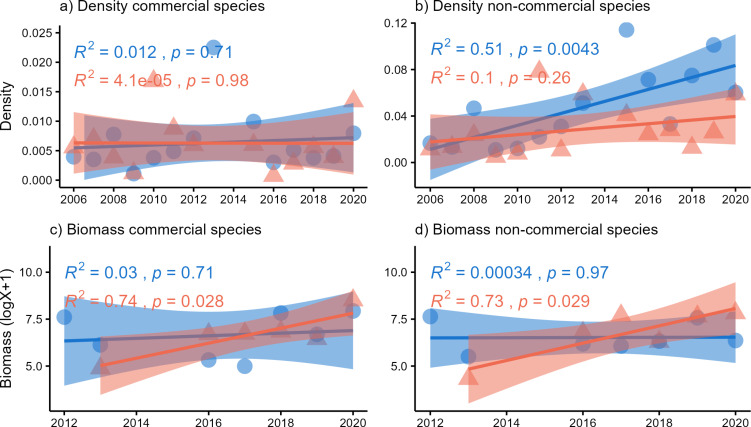
The temporal trends for density (a and b) and biomass (c and d) of commercial and non-commercial fish species at protected (MUMPA: blue) and non-protected (NP: orange) sites of the Oaxaca coast are presented. The linear model indicators, R^2^ and P, are displayed alongside each graph.

Regression analyses for MUMPA sites indicated an increase in the density of non-commercial fish, while the density of commercial species and the biomass of all the species did not change significantly over time. In contrast, there was no change in fish density at NP sites, while there was a significant increase in biomass for both commercial and non-commercial species from 2012 to 2020. Consequently, while reef fish biomass outside the MUMPA exhibited an increase, it remained stable within it throughout the study period (Fig 4).

One-way SIMPER analysis of density revealed a high dissimilarity percentage (61%) between MUMPA and NP sites, with the former showing a higher density of *Thalassoma lucasanum, Chromis atrilobata*, *Apogon pacificus,* and *Stegastes acapulcoensis* (Table 1). The high density of these species, which exhibit typical reef fish traits (e.g., small to medium size, sedentary, and bentho-pelagic), are the results of the temporal increase of non-commercial species at MUMPA sites described previously (Fig 4b).

**Table 1:** The species that contributed to the dissimilarity (Bray-Curtis coefficient) in density among the MUMPA and NP sites of the Oaxaca coast according to the SIMPER analysis (70% cut-off) are presented in the following table. Commercial species are indicated with an asterisk (*).

Density	Average dissimilarity = 61%		
	MUMPA sites	NP sites	
Species	Av. Density	Av. Density	Contrib%
*Thalassoma lucasanum*	0.83	0.54	21.37
*Chromis atrilobata*	0.42	0.30	14.86
*Apogon pacificus*	0.53	0.09	14.70
*Stegastes acapulcoensis*	0.40	0.29	9.64
Haemulon maculicauda*	0.09	0.20	7.30
*Halichoeres dispilus*	0.11	0.03	3.96

Concerning biomass, the dissimilarity percentage between the MUMPA and NP sites was 74% (Table 2), which was associated with a higher biomass of commercial medium to large-sized (> 30 cm) piscivores, such as *Caranx caballus* and *Cephalopholis panamensis* at the MUMPA sites. Furthermore, the NP sites exhibited a dominance of large (> 50 cm) commercial piscivores (*Haemulon sexfasciatum*) and non-commercial herbivores (*Prionurus laticlavius* and *Acanthurus xanthopterus*). The observed increase in medium and large-sized fish (> 30 cm) could explain the overall increase in biomass at NP sites, as illustrated in the regression analysis (Fig 4c and 4d).

**Table 2:** The species that contributed to the dissimilarity (Bray-Curtis coefficient) in biomass among the MUMPA and NP sites of the Oaxaca coast are presented herewith, according to the SIMPER analysis (70% cut-off). Commercial species are indicated by an asterisk (*).

Biomass	Average dissimilarity = 74%		
	MUMPA sites	NP sites	
Species	Av. Biomass	Av. Biomass	Contrib%
*Prionurus laticlavius*	355.62	1668.31	23.02
*Stegastes acapulcoensis*	767.08	462.50	11.55
*Acanthurus xanthopterus*	2.47	397.85	6.52
Caranx caballus*	274.89	39.91	6.30
Cephalopholis panamensis*	399.95	21.88	5.94
*Halichoeres nicholsi*	333.07	19.85	4.92
*Arothron meleagris*	195.61	193.86	4.50
Haemulon sexfasciatum*	3.78	411.46	4.18
*Microspathodon dorsalis*	255.13	184.27	3.51

## Discussion

A total of 84 conspicuous fish species were identified on the Oaxaca coast, consistent with previous regional studies that reported a range of 60–89 species [23,24]. The comparison between human use levels confirmed the resemblance in the fish diversity hypothesis, with slightly higher values at MUMPA sites (78 species) in contrast to NP zones (73 species). This is consistent with previous studies on MUMPAs worldwide, which have demonstrated similar values of taxonomic metrics than fishing zones attributed to the persistence of economic activities (e.g., fishing and tourism) within their protection polygons [12].

In addition to the resemblance in species richness, we observed similarities in the total number of FEs among MUMPA (65 FEs) and NP (63 FEs) sites. This contrasts with the results from Hernandez-Andreu et al. [52] who found that tropical MPAs presented lower values of S and FE than NP zones, suggesting that the level of protection does not necessarily safeguard the functionality of reef ecosystems. In our case, the similar values observed in the ecological indicators exhibited resemblance in the functionality of ecosystems at both human use levels, which can be attributed to several factors, including the design and management of the MUMPA [11]. Studies of well-designed and effective managed MUMPAs have reported greater stability in some aspects of biodiversity [53,54], as well as the expansion of benefits to harvesting areas through the dispersal of larvae and the propagation of juveniles and adults outside the protection polygon [55]. Nonetheless, further investigation into these topics is necessary at Parque Nacional Huatulco, in order to provide direct evidence the effects of protection inside and outside the MUMPA.

The temporal analysis showed that species richness and density increased significantly at the MUMPA and NP sites, while species dominance remained consistent from 2006 to 2020. The increase and maintenance of biodiversity could be considered benefits of protection since the aim of Parque Nacional Huatulco is to preserve biodiversity and to promote its conservation through the sustainable use of these resources [28]. Notably, the only site that has shown results similar to our findings is Parque Nacional Cabo Pulmo, which is considered the only fully protected area in México and has witnessed a considerable surge in fish species richness and density, attributed to the confluence of social and ecological factors [16]. The region’s robust social fabric, characterized by effective self-enforcement by local stakeholders and broad community support, has played a pivotal role. Additionally, a sizable core zone, a well-conserved coral reef, the presence of fish spawning aggregations, and high primary productivity have collectively contributed to the remarkable enhancement of marine biodiversity [16]. Similarly, the sites within Parque Nacional Huatulco are regarded as among the most well-preserved along the Pacific coastline. This is likely attributable to the limited human impact on these sites, as the majority of them are only accessible by boat, thereby allowing them to remain in favorable conditions that support high reef fish biodiversity [30].

We can draw inferences about ecological factors that may account for the observed increase in these indicators at Parque Nacional Huatulco, such as the maintenance of coral cover throughout the study [30] and the high primary productivity resulting from upwelling events in the region [20]. Nonetheless, the social factors (e.g., MPA zonation, surveillance efforts, local stakeholder participation, enforcement of environmental regulations) that underpin ecosystem-based management actions at this MUMPA warrant further investigation since protected sites only showed slightly higher estimated values compared to NP sites. Moreover, data regarding visitor numbers and fishing pressure at the MUMPA are required to ascertain the dynamics of economic activities and their impact on fish diversity.

The maintenance of Simpson’s dominance throughout the study period provides an additional illustration of the effects of local conservation initiatives. This suggests that taxonomic homogenization (typically associated with harvesting) is not a significant factor in the region, which aligns with the finding in the Mediterranean reefs, where a disproportionate impact of protection on rare and common species has been documented [56]. The resemblance among MUMPAs and fishing zones has also been interpreted as indicating no social or ecological benefits from protection [15].

Our findings differ from these results, since the observed increases or stability in taxonomic and functional indices within the MUMPA and NP sites, could indicate that the socio-ecological conditions in the study area, in addition to the management strategies employed at Parque Nacional Huatulco, appear to contribute to the conservation of fish assemblages along the coastline. Given that the positive effects of MUMPAs can be enhanced by the declaration of adjacent fully protected areas, establishing larger zones dedicated only to conservation activities and scientific research should be considered for future management program updates [14,15].

Significant changes were observed in FRic, FDiv, and FOri in MUMPA sites, whereas these indices remained stable over time in NP sites. Although temporal variation could be attributed to methodological factors (e.g., time-of-day, tidal state, distinct divers), our study design allowed us to minimize these effects by keeping these variables as consistent as possible over the time series. Furthermore, intra-annual variation and spatial heterogeneity were incorporated as random factors in the LMMs, since these variables showed significant effects on the ecological indicators. This allowed us to circumvent any potential confounding effects and isolate the factor under investigation, namely the change in fish diversity over the study period [47,57,58]. Nonetheless, differences in sampling units throughout the years, sites, and human use levels, respond to funding and resource availability, which are often focused on in the study of MUMPAs. Based on this, we consider necessary to increase sampling efforts outside the protection polygons, to determine the effectiveness of MUMPAs in the conservation of fish assemblages in this and other regions of the Mexican Pacific.

Concerning functional diversity, we observed that while NP sites demonstrated stability over time, MUMPA sites exhibited increases in FRic and decreases in FOri. The positive shift in FRic within protected sites suggests an increase of species with extreme traits that theoretically perform different functions compared to species typically found on reefs [35]. However, this increase in FRic does not necessarily indicate substantial changes in reef processes over time. Indeed, density increases were concentrated among species located near the centroid of the functional space, which includes those with typical reef fish traits such as small to medium size, low mobility, and benthic association. This pattern is further reflected in the significant decrease in FOri (opposite of functional redundancy) and FDiv, which indicates the proportion of total density supported by FEs with extreme traits [26,35]. Moreover, the high contribution of species such as *T. lucasanum*, *C. atrilobata*, and *S. acapulcoensis* to the aggregation of MUMPA sites lends support to the notion that protection at Parque Nacional Huatulco is conducive to the proliferation of common species, thereby enhancing functional redundancy and the capacity to sustain typical reef fish functions over time [26,35,59].

The fish diversity increases observed in this tropical region in contrast with the declines previously reported in MUMPAs of the Gulf of California [18]. Consequently, our initial hypothesis of a decrease in fish taxonomic and functional diversity along the Oaxaca coast was rejected. Furthermore, the increases in fish species richness and density were slightly higher in MUMPA sites compared to NP sites, suggesting potential benefits of restrictions on human activities (e.g., fishing and tourism) in maintaining fish diversity, even under low to moderate natural disturbance regimes. Thus, could be necessary to implement measures restricting human activities to conserve fish diversity in the face of low to moderate-intensity natural disturbances. As increases in species density and biomass have been linked to particular species that rapidly respond to temperature anomalies, temporal changes in fish diversity may also be caused by environmental conditions; for example temperature rise can lead to the movement of fish, particularly migratory species, resulting in increased abundances of adults and ichthyoplankton [18,60]. In this context, we have also observed significant inter-annual variation in fish species richness, density, and FRic, with the highest values recorded during the 2015–2016 El Niño–Southern Oscillation (ENSO) event, when coral mortality remained modest [30]. Similar increases in these ecological indicators have previously been attributed to the influx of tropical species due to the incursions of warm waters during low to moderate-intensity ENSO events in the Eastern Tropical Pacific [61]. Nevertheless, further investigation is required to fully assess the long-term impacts and evaluate the consequences of such events, since our analysis did not detect an effect of the 2009–2010 event. Significant reductions in fish biomass and other reef-associated fauna (echinoderms) were reported during the summer of 2023 at Parque Nacional Huatulco compared to the period between 2020 and 2021 [30,62]. In their analysis, the authors identified that corallivorous (e.g., *Pseudobalistes naufragium*, *Cantherhines dumerilii*, *Arothron hispidus*) and carnivorous species (e.g., *Haemulon scudderii*, *Caranx caninus*) were the most affected by the coral degradation (90% of the shallow *Pocillopora* colonies presented coral bleaching) [30]. This led to a general decrease in the average trophic level from 3.43 in 2020–2021 to 3.28 in 2023 [30]. In addition, prior studies conducted along the Oaxaca coast have documented a decline in fish diversity attributed to coral reef degradation and overfishing. As a result, the abundance of commercially important families, including Lutjanidae, Serranidae, and Carangidae have been reduced [19].

Our results demonstrated a rise in commercial fish biomass at NP sites, appears to be linked to an increase in average fish size since the density remained stable throughout the study period. The increase in average fish size is typically considered a direct effect of protection, reflecting the return or growth of commercial species due to reduced fishing pressure [26]. However, commercial species exhibited biomass stability at MUMPA sites, thus, this result could be attributed to ecological factors (e.g., availability of resources and adequate physical conditions) rather than direct benefits of protection [63]. Consequently, the hypothesis of a decline in commercial species was rejected. Furthermore, the SIMPER analysis for biomass demonstrated that large-sized piscivores made significant contributions to fish assemblages. These contributions were particularly evident in the aggregation of MUMPA (*C. caballus* and *C. panamensis*) and NP (*H. sexfasciatum*) sites, emphasizing the presence of commercial species and their provisioning services at both zones.

Non-commercial species also showed increases or stability in biomass and density across both human use levels, consistent with the reported dominance of families such as Pomacentridae and Labridae in the study area [19]. The observed increase in non-commercial species, usually generalist, might be associated with coral reef degradation and the cover of alternative substrates, including sand, dead coral, and macroalgae increase [19]. The degradation of coral reef habitats and the emergence of generalist fish assemblages at a regional scale could be an effect of different natural and anthropogenic factors, such as sea temperature anomalies, hurricanes, and intensified human activities [64,65]. Thus, it is recommended that coral reef restoration and habitat conservation efforts be given priority at Parque Nacional Huatulco, to ensure the preservation of essential habitats and to promote the maintenance of fish functions for the coming decades.

In terms of human use, the Official Council of Tourism has reported an increase in visitors to the Huatulco region, which could potentially exert a detrimental impact on the environment due to the physical damage that tourism activities such as boat traffic, snorkeling, and diving can cause to coral colonies and the associated reef-fish fauna [66]. Despite the prohibition of direct interactions with marine organisms (e.g., feeding, chasing, harassing, bothering, or removing) at Parque Nacional Huatulco [28], the practice of feeding Labrids and other non-commercial species is a common occurrence among snorkel guides. The observed increase in these activities could explain the increment in non-commercial species, which could be attributed to these activities (personal communication), particularly given that certain groups —such as herbivores, planktivores, and invertivores— are known to exhibit rapid changes following disturbance [13,54]. To mitigate potential impacts, it is necessary to promote sustainable practices within the tourism sector and continue monitoring these species, to enable swift responses to shifts in human activities and ecological conditions.

## Conclusions

Our hypothesis regarding the resemblance in fish diversity among human use levels was accepted, as evidenced by the similarity in taxonomic and functional diversity, which can be attributed to several factors, including the design and management of the MUMPA, and the possible expansion of benefits to NP sites. Furthermore, the study revealed a surge in species richness and fish density throughout the investigation with slightly higher positive changes on protected sites. These trends may be linked to ecological factors such as the maintenance of coral cover and high primary productivity in the region. However, further investigation is necessary to determine the social factors that support ecosystem-based management actions at Parque Nacional Huatulco. Moreover, while fish functional diversity remained stable at NP sites, MUMPA sites exhibited positive changes in FRic, indicating an increase in the presence of species with extreme traits, and declines in FOri and FDiv driven by increases in the density of common reef species. These findings support the notion that the conservation measures at Parque Nacional Huatulco are conducive to a fish diversity increase, thereby enhancing the capacity to maintain common reef fish functions over time. Both commercial and non-commercial species demonstrated stability or increases in density and biomass at both human use levels, despite the natural and anthropogenic events that threatened reef ecosystems within Parque Nacional Huatulco during the study period. These events included the 2009–2010 and 2015–2016 ENSO events and the associated coral reef degradation. Based on the results of our study, we recommend that the monitoring program be continued, that the protected zones be expanded, and that coral habitat restoration actions be promoted, to conserve coral habitats. In addition, we suggest that sustainable practices be enhanced within the tourism sector to encourage the maintenance of fish functions for the coming decades.

## Supporting information

S1 Text Supporting information: Conservation of Fish Diversity in Protected Sites and Adjacent Fishing Areas of the Southern Mexican Pacific Ocean. This document contains Tables A-E and Figures A-D.
(DOCX)
