# Supplementary material for: Conservation of fish diversity in protected sites and adjacent fishing areas of the Southern Mexican Pacific Ocean
Source: PLoS One. 2025 Jun 4;20(6):e0324155. doi: 10.1371/journal.pone.0324155 (PMC12136324; doi:10.1371/journal.pone.0324155)
Supplement: S1 Text — This document contains Tables A-E and Figures A-D. (DOCX) [file pone.0324155.s001.docx]

**Supporting information: Conservation of Fish Diversity in Protected Sites and Adjacent Fishing Areas of the Southern Mexican Pacific Ocean**

Georgina Ramírez-Ortiz^1¶^, Omar Valencia-Méndez^2¶^, Luis Hernández^3&^, Tania González-Mendoza^2&^, Andrés López-Pérez^4&^.

^1^ Laboratorio de Ecología funcional & conservación marina, Instituto de Ciencias del Mar y Limnología, Universidad Nacional Autónoma de México, Mazatlán, Sinaloa, México.

^2^ Laboratorio de Esclerocronología, Ecología y Pesquerías de la Zona Costera, Departamento de Ecología Marina, Centro de Investigación Científica y de Educación Superior de Ensenada, Ensenada, Baja California, México.

^3^ Laboratorio de Sistemas arrecifales, Departamento Académico de Ciencias Marinas y Costeras, Universidad Autónoma de Baja California Sur, La Paz, Baja California Sur, México.

^4^ Laboratorio de Arrecifes y Biodiversidad/Ecosistemas Costeros, Departamento de Hidrobiología, Universidad Autónoma Metropolitana-Iztapalapa, Iztapalapa, Ciudad de México, México.

Corresponding author

E-mail: [gramirezortiz@ola.icmyl.unam.mx](mailto:gramirezortiz@ola.icmyl.unam.mx)

**Table A.** The sampling units per year and sites performed at the protected and non-protected sites of the Oaxaca coast within the study period (2006-2020) are presented.

| **Site** | **2006** | **2007** | **2008** | **2009** | **2010** | **2011** | **2012** | **2013** | **2015** | **2016** | **2017** | **2018** | **2019** | **2020** | **Sampling units per site** |
| --- | --- | --- | --- | --- | --- | --- | --- | --- | --- | --- | --- | --- | --- | --- | --- |
| **Multi-use Marine Protected Area (MUMPA)** | | | | | | | | | | |  |  |  |  | 627 |
| Cacaluta | 79 | 10 | 4 | 4 | 4 | 8 | 4 | 6 | 3 | 4 | 4 |  | 4 | 6 | 140 |
| Dos_Hermanas | 60 | 10 |  |  |  | 2 |  |  |  |  | 4 |  | 4 | 6 | 86 |
| India |  |  |  |  |  | 4 |  |  |  |  | 4 |  | 4 | 4 | 16 |
| Isla_San_Agustín | |  | 4 |  |  | 4 | 4 | 5 | 3 |  | 4 |  |  | 4 | 28 |
| Jicaral | 70 | 10 |  |  |  |  | 4 | 8 | 3 | 5 | 4 |  | 4 | 4 | 112 |
| Maguey |  |  | 7 | 4 |  | 2 |  |  | 3 | 3 | 4 | 4 | 4 | 4 | 35 |
| Órgano |  |  |  |  |  | 4 |  |  |  |  | 3 |  |  | 4 | 11 |
| Riscalillo |  |  | 7 | 4 | 4 | 8 | 4 | 8 | 3 | 4 | 4 |  | 4 | 4 | 54 |
| San_Agustín | 65 | 10 |  | 4 |  | 4 | 4 | 8 | 3 | 4 | 6 |  | 4 | 4 | 116 |
| Violín |  |  | 3 | 4 |  | 3 | 2 | 2 | 3 |  | 4 | 4 |  | 4 | 29 |
| **Non-protected sites (NP)** | | |  |  |  |  |  |  |  |  |  |  |  |  | 328 |
| Boquilla |  |  |  |  |  |  |  |  |  |  |  |  |  | 4 | 4 |
| Chahue |  |  |  |  |  |  |  |  |  |  | 4 |  |  | 4 | 8 |
| Entrega | 80 | 10 | 6 | 2 | 4 | 8 | 2 | 7 | 3 | 4 | 6 | 8 | 4 | 8 | 152 |
| Estacahuite |  |  |  |  |  |  |  |  |  |  |  |  |  | 4 | 4 |
| Manzanilla |  |  | 4 |  |  |  |  |  |  |  |  |  |  | 4 | 8 |
| Mazunte |  |  |  |  |  |  |  | 4 |  |  |  |  |  | 4 | 8 |
| Mina |  |  |  |  |  |  |  |  |  |  |  |  |  | 4 | 4 |
| Montosa | 80 | 10 | 3 | 6 | 4 | 4 |  |  | 3 |  | 4 |  |  | 4 | 118 |
| Salchi |  |  |  |  |  |  |  |  |  |  |  |  |  | 4 | 4 |
| Tijera |  |  |  |  |  |  |  | 4 |  |  |  |  |  | 4 | 8 |
| **Sampling units per year** | 434 | 60 | 38 | 28 | 16 | 51 | 24 | 52 | 27 | 24 | 55 | 16 | 32 | 88 |  |
|  |  |  |  |  |  |  |  |  |  |  |  |  |  |  |  |


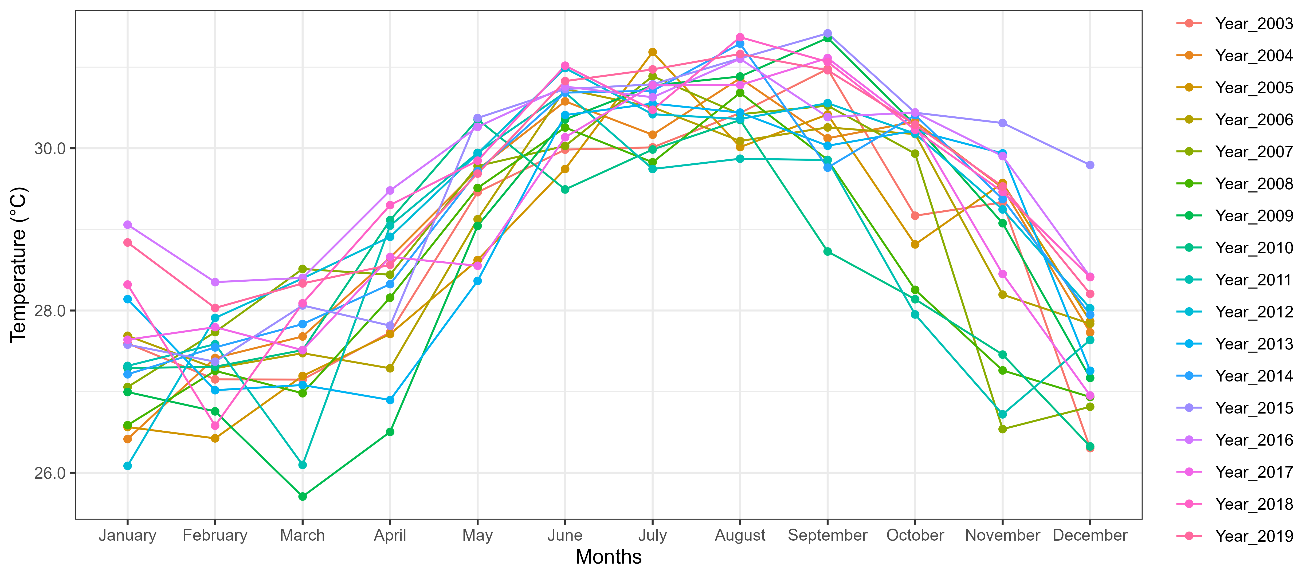


**Figure A.** Historical sea surface temperature data for Parque Nacional Huatulco, gathered from monthly satellite images from 2003 to 2019 using the Moderate Resolution Imaging Spectroradiometer (MODIS) Aqua sensor.


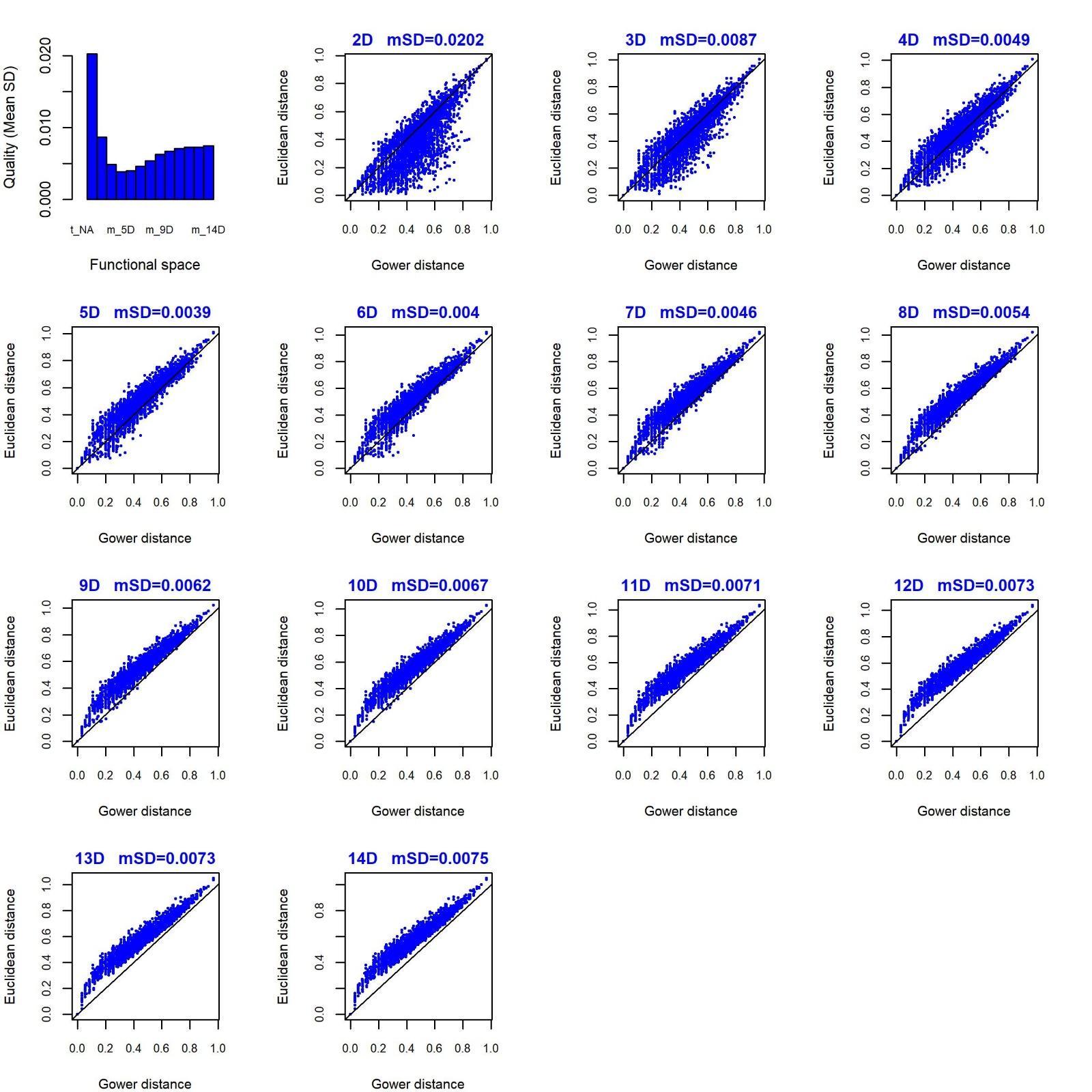


**Figure B.** Quality of the functional space graph performed to define the number of dimensions used to calculate the functional indices in the present study. In this case, we used five dimensions since the mean squared deviation between the initial functional distances (i.e. based on traits) and the scaled functional distances in the functional space were < 0.005, and after that, the number of dimensions increased (Maire et al. 2015).

**
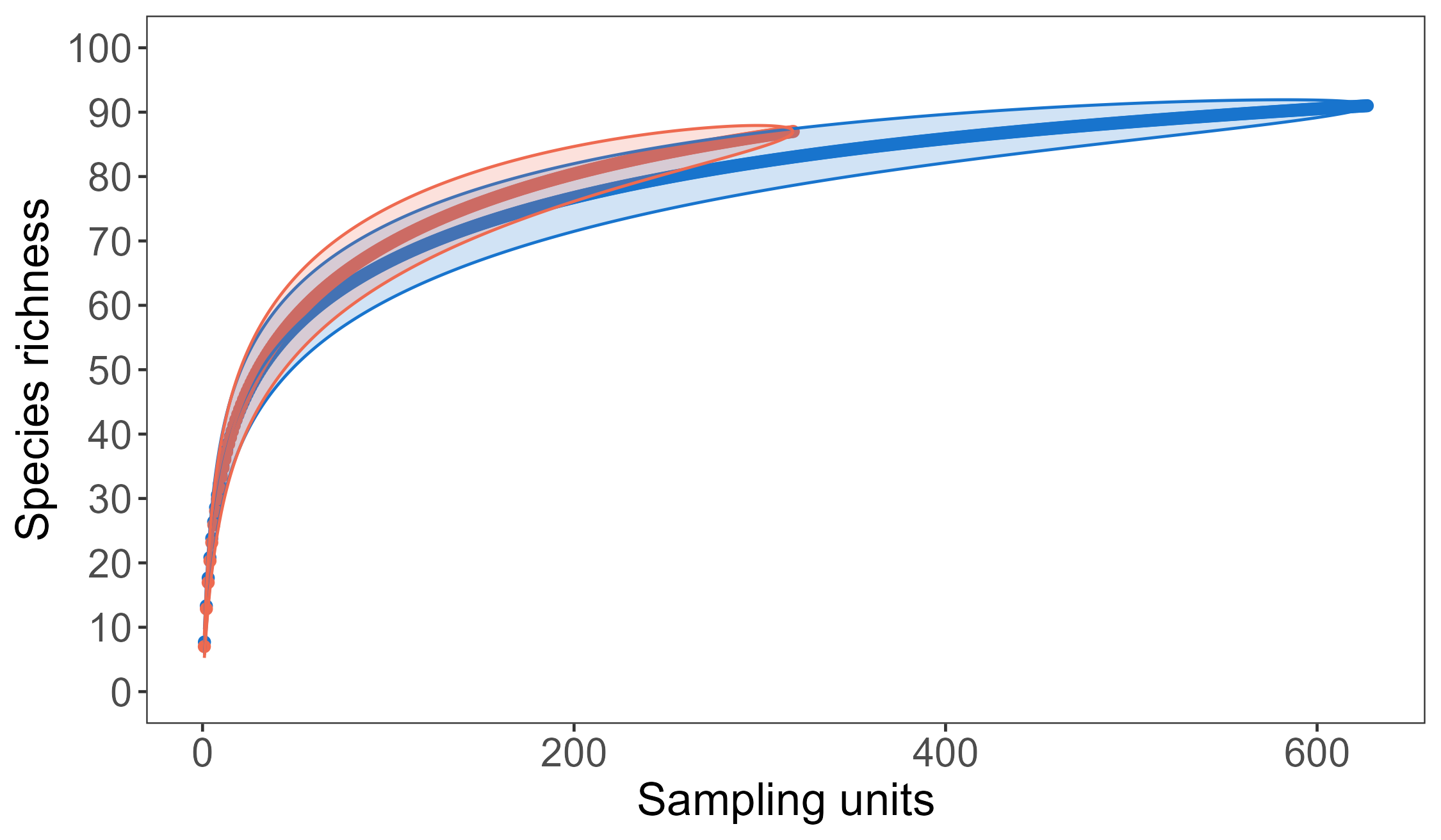
Figure C.** Rarefaction curves by human use levels: multi-use Marine Protected Area in blue and Non-protected sites in red. The independent variable is the number of sampling units and the dependent variable is the number of species.

**Table B.** Biological traits considered for the fish functional analysis at protected and non-protected sites on the Oaxaca coast.

|  |  |  |  |  |  |  |
| --- | --- | --- | --- | --- | --- | --- |
| **Ordinal** | **1** | **2** | **3** | **4** | **5** | **6** |
| Maximum  fish size (cm) | 3-7 | 7.1-15 | 15.1-30 | 30.1-50 | 50.1-80 | > 80 |
| Mobility | Sedentary | Mobile within-reef | Mobile among reefs | Very mobile with an extensive home range |  |  |
| Gregariousness | Solitary | Pairing | Small group (3-50 ind) | Large group (> 50 ind) |  |  |
| Position in the water column | Benthic | Bentho-pelagic | Pelagic |  |  |  |
| **Nominal** |  |  |  |  |  |  |
| Period of activity | DiurnaL (D) | Nocturnal (N) |  |  |  |  |
| Diet | Herbivore-Detritivore (HD) | Invertivore targeting sessile invertebrates (IS) | Invertivore targeting mobile invertebrates (IM) | Planktivorous (Pk) | Piscivore (FC) | Omnivore (Om) |

**Table C.** Protection analyses of fish diversity at the Oaxaca coast, based on LMMs (Index ~ human use level + (1|year) + (1|site) + (1|season)) for six ecological indicators: species richness (S), density (d (log_2_)), Simpson’s dominance index (D), functional richness (FRic), functional divergence (FDiv), and functional originality (FOri). P> 0.05 indicates a non-significant effect of protection for fish ecological indicators, while temporal, spatial and seasonal effect is indicated by the random standard deviation (RSD).

| **Index** | **Intercept** | **Estimate** | **Standard error** | **T** | **P** | **RSD Year** | **RSD Site** | **RSD Season** |
| --- | --- | --- | --- | --- | --- | --- | --- | --- |
| S | 10.82 | 0.48 | 0.70 | 0.69 | 0.50 | 1.73 | 1.32 | 0.19 |
| d (log_2_) | 0.78 | -0.02 | 0.24 | -0.08 | 0.93 | 0.89 | 0.39 | 0.07 |
| D | 0.63 | 0.00 | 0.03 | 0.09 | 0.93 | 0.04 | 0.04 | 0.03 |
| FRic | 0.02 | 0.00 | 0.01 | -0.51 | 0.62 | 0.02 | 0.01 | 0.00 |
| FDiv | 0.78 | 0.02 | 0.01 | 1.11 | 0.29 | 0.09 | 0.02 | 0.04 |
| FOri | 0.16 | 0.02 | 0.01 | 1.95 | 0.07 | 0.02 | 0.02 | 0.01 |

**Table D.** Temporal analyses of fish diversity at the Oaxaca coast, based on LMMs (Index ~ year + (1|site) + (1|season)) for six ecological indicators: species richness (S), density (d (log_2_)), Simpson’s dominance index (D), functional richness (FRic), functional divergence (FDiv), and functional originality (FOri). P< 0.05 indicates a significant temporal effect for fish ecological indicators (in bold), while the spatial and seasonal effect is indicated by the random standard deviation (RSD).

| **Index** | **Intercept** | **Estimate** | **Standard error** | **T** | **P** | **RSD Site** | **RSD Season** |
| --- | --- | --- | --- | --- | --- | --- | --- |
| **Multi-use Marine Protected Area (MUMPA)** | | | | | | |  |
| S | 9.20 | 0.17 | 0.04 | 4.82 | **< 0.01** | 1.18 | 0.07 |
| d (log_2_) | -0.61 | 0.17 | 0.02 | 11.08 | **< 0.01** | 0.38 | < 0.01 |
| D | 0.61 | < 0.01 | < 0.01 | -1.74 | 0.08 | 0.04 | 0.03 |
| FRic | 0.02 | < 0.01 | < 0.01 | 2.34 | **0.02** | 0.01 | < 0.01 |
| FDiv | 0.81 | < 0.01 | < 0.01 | -2.92 | **< 0.01** | 0.02 | 0.04 |
| FOri | 0.18 | < 0.01 | < 0.01 | -3.00 | **< 0.01** | 0.02 | < 0.01 |
| **Non-protected sites (NP)** | | | | | | |  |
| S | 9.54 | 0.11 | 0.04 | 2.57 | **0.01** | 1.30 | 0.34 |
| d (log_2_) | -0.36 | 0.11 | 0.02 | 4.60 | **< 0.01** | 0.47 | < 0.01 |
| D | 0.58 | < 0.01 | < 0.01 | 0.48 | 0.63 | 0.04 | 0.01 |
| FRic | 0.01 | < 0.01 | 1.18 | 19.16 | 0.25 | < 0.01 | < 0.01 |
| FDiv | 0.84 | < 0.01 | < 0.01 | -1.53 | 0.14 | < 0.01 | < 0.01 |
| FOri | 0.19 | < 0.01 | < 0.01 | -0.93 | 0.36 | 0.03 | < 0.01 |

**Table E.** Statistical comparisons of fish ecological indicators between years in Parque Nacional Huatulco (MUMPA) and contiguous fishing sites (NP) using ANOVA. P values in bold letters indicate significant differences. Species richness (S), density (d (log2)), Simpson’s dominance index (D), functional richness (FRic), functional divergence (FDiv), and functional originality (FOri).

| **Index** | **Mean Sq** | **F** | **P** | **Years with a significant difference** |
| --- | --- | --- | --- | --- |
| **Marine Protected Area (MUMPA)** | | | |  |
| S | 143.456 | 18.984 | **< 0.001** | 2006-2008, 2010-2013, 2015-2020 |
| d (log2) | 42.992 | 27.564 | **< 0.001** | 2006, 2008, 2011-2013, 2015-2020 |
| D | 0.094 | 2.813 | **< 0.001** | 2006, 2012, 2013, 2020 |
| FRic | 0.01 | 15.679 | **< 0.001** | 2006-2008, 2011-2013, 2015-2018 |
| FDiv | 0.079 | 6.498 | **< 0.001** | 2006-2009, 2011, 2012, 2015, 2017, 2018 |
| FOri | 0.008 | 2.409 | **0.003** | 2006, 2016, 2020 |
| **Non-protected sites (NP)** | | | |  |
| S | 29.128 | 4.822 | **< 0.001** | 2006, 2011, 2013, 2015, 2020 |
| d (log2) | 16.834 | 9.293 | **< 0.001** | 2006, 2008, 2011, 2013, 2015, 2017, 2019, 2020 |
| D | 0.037 | 1.119 | 0.342 | 2006 |
| FRic | < 0.001 | 2.889 | **< 0.001** | 2006, 2011, 2013, 2015 |
| FDiv | 0.02 | 1.816 | **0.041** | 2006, 2011, 2020 |
| FOri | 0.006 | 2.449 | **0.003** | 2006, 2008, 2010, 2011 |

**
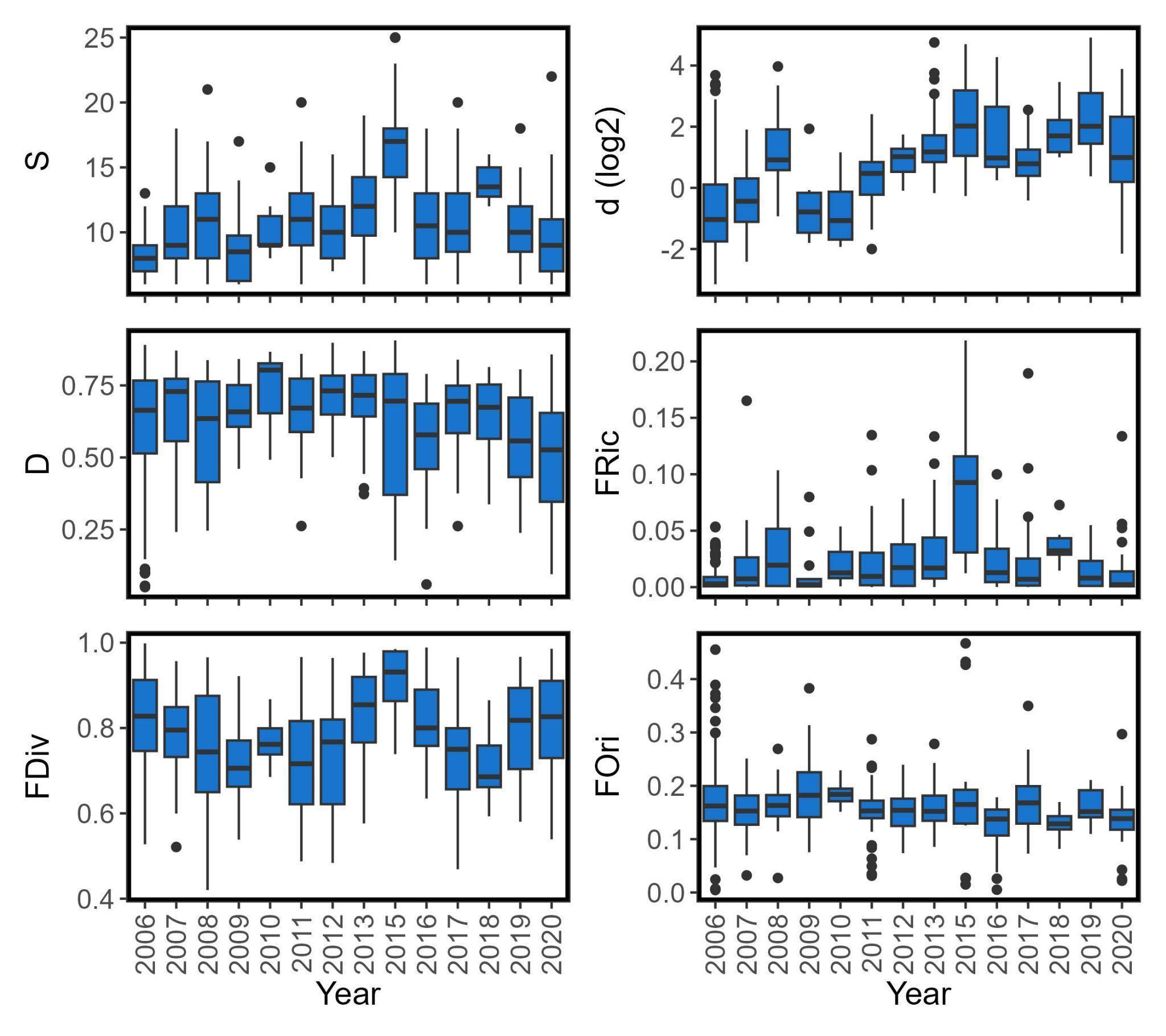
**

**Figure D.** Comparison of fish diversity between years in the protected sites of Oaxaca coast. The boxplots depict the mean, 1st and 3rd quartiles, the confidence interval (95%), and the outlier dots. Species richness (S), density (d (log2)), Simpson’s dominance index (D), functional richness (FRic), functional divergence (FDiv), and functional originality (FOri).

**
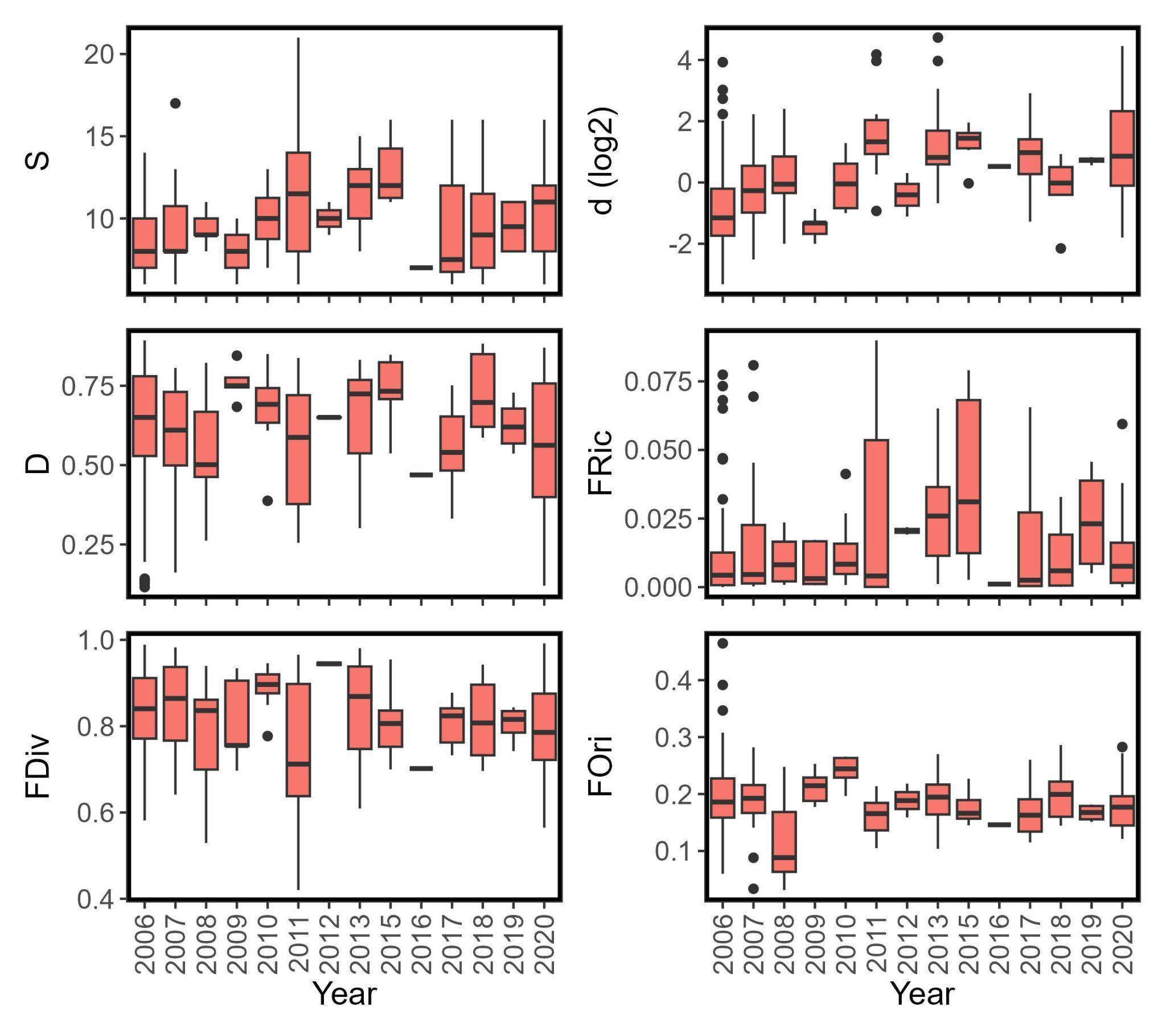
**

**Figure E.** Comparison of fish diversity between years in the non-protected sites of Oaxaca coast. The boxplots depict the mean, 1st and 3rd quartiles, the confidence interval (95%), and the outlier dots. Species richness (S), density (d (log2)), Simpson’s dominance index (D), functional richness (FRic), functional divergence (FDiv), and functional originality (FOri).

**Reference**

[Maire E, Grenouillet G, Brosse S, Villéger S. How many dimensions are needed to accurately assess functional diversity? A pragmatic approach for assessing the quality of functional spaces. Glob Ecol Biogeogr. 2015;24: 728–740. doi:10.1111/geb.12299](https://www.zotero.org/google-docs/?KLlCCF)
